# A Novel Method to Identify Autoantibodies against Putative Target Proteins in Serum from beta-Thalassemia Major: A Pilot Study

**DOI:** 10.3390/biomedicines8050097

**Published:** 2020-04-26

**Authors:** Afshan Sumera, Nur Diana Anuar, Ammu Kutty Radhakrishnan, Hishamshah Ibrahim, Nurul H. Rutt, Nur Hafiza Ismail, Ti-Myen Tan, Abdul Aziz Baba

**Affiliations:** 1 School of Medicine, International Medical University, Bukit Jalil, Kuala Lumpur 57000, Malaysia; abdulazizbaba@imu.edu.my; 2 Sengenics Corporation Pte Ltd., Singapore 409051, Singapore; n.diana@sengenics.com (N.D.A.); n.rutt@sengenics.com (N.H.R.); n.hafiza@sengenics.com (N.H.I.); t.myen@sengenics.com (T.-M.T.); 3 Jeffrey Cheah School of Medicine and Health Sciences, Monash University Malaysia, Sunway 47500, Malaysia; ammu.radhakrishnan@monash.edu; 4 Paediatrics Department, Kuala Lumpur General Hospital, Jalan Ipoh, Kuala Lumpur 50586, Malaysia; drhishamshah@moh.gov.my

**Keywords:** thalassemia, autoantibodies, globin gene, immune response, biomarkers

## Abstract

Abnormal immune reactivity in patients with beta-thalassemia (beta-thal) major can be associated with poor prognosis. Immunome protein-array analysis represents a powerful approach to identify novel biomarkers. The Sengenics Immunome Protein Array platform was used for high-throughput quantification of autoantibodies in 12 serum samples collected from nine beta-thal major patients and three non-thalassemia controls, which were run together with two pooled normal sera (Sengenics Internal QC samples). To obtain more accurate and reliable results, the evaluation of the biological relevance of the shortlisted biomarkers was analyzed using an Open Target Platform online database. Elevated autoantibodies directed against 23 autoantigens on the immunome array were identified and analyzed using a penetrance fold change-based bioinformatics method. Understanding the autoantibody profile of beta-thal major patients would help to further understand the pathogenesis of the disease. The identified autoantigens may serve as potential biomarkers for the prognosis of beta-thal major.

## 1. Introduction

Thalassemia is the most commonly inherited single-gene disorder in the world, with an overall carrier frequency of 2%–25% [1]. In Malaysia, the carrier rate is about 4.5% [2] amongst the Malays and Chinese [3]. Beta-thalassemia (beta-thal) major is a disorder caused by mutations in the *β-globin* gene, which can result in total absence of β-globin chains. The deficiency of β-globin chains causes an imbalance in the ratio between α- and β-globin chains within mature and immature erythrocytes [4]. Unpaired α-globin chains are very toxic to erythrocytes, as they can liberate reactive iron and induce oxidative stress to the plasma membrane and proteins within these cells. Excess α-globin chains also tend to increase intracellular calcium levels, which exposes phosphatidylserine residues; which can result in early apoptosis in the affected red blood cells (RBC). Beta-thal major manifests by ineffective erythropoiesis, peripheral hemolysis and, eventually, anemia [4].

In the past, conventional proteomics methods used were not sensitive enough to detect RBC cytosol and skeletal membrane proteins due to high abundance of hemoglobin (Hb) in these samples. Recently, with the availability of novel Hb depletion methods and more robust detection strategies, the protein profiling of erythrocytes is possible. Understanding the inter-relationships between the RBC membrane and cytosolic proteins affected by disease processes can help to provide more information about the pathophysiological mechanisms of various diseases [5]. In 2013, researchers who studied differences between RBC proteins in normal vs. sickle cell disease reported that RBC proteins cluster into a functional domain cluster, which they named the “repair or destroy box’’ [6]. Twenty differentially expressed proteins between normal and sickle cell disease were identified. In addition, the major mechanisms causing the pathology were identified to be continuous oxidative stress, which caused damage to cytosolic and membrane proteins of RBC [7]. The first proteomics study in beta-thal patients with hemoglobin E (HbE) disease was conducted using 2D gel electrophoresis followed by MALDI-MS/MS tandem mass spectrometry, where the authors identified seven differentially expressed proteins that were either up or downregulated [8]. In addition, they found 12 RBC cytosolic proteins that were differentially expressed between normal subjects and beta-thal/HB-E patients. In another study on plasma proteome profile of beta-thal/HB-E to assess the severity of the disease, a list of differentially expressed plasma proteins between healthy subjects versus beta-thal/HB-E patients and mild vs. severe forms of beta-thal/HB-E were identified [9]. These proteins may serve as good candidates for further studies on the pathophysiology of beta-thal/HB-E or for biomarkers to define the severity of beta-thal/HB-E. 

A protein microarray is a miniaturized technology, currently evolving, and is used for many complex biomarker discoveries in oncology, molecular and immunotherapeutic pathways [10]. In beta-thal major, immune abnormalities are not uncommon and can result in organ dysfunction [11]. Considering this fact, the identification of possible autoantibodies by protein microarray technology in principle can augment the detection of the humoral immune response in beta-thal major. Currently, different types of protein microarrays are available. The immunome protein microarray utilizes the KREX technology, which is highly sensitive and can be used to detect antigen-specific antibodies in various conditions [12]. This technique is also helpful in quantifying autoantibodies as well as in comparing the results across available datasets using a functional examination of target proteome subsets systematically, the quantitation of reaction and technical consistency [13]. Several studies have shown that this technique is valuable for the discovery of biomarkers in many diseases [14,15,16].

There is a lack of literature on the expression patterns and analysis of potential serological biomarkers for disease severity in beta-thal major. The current study is a pilot study, which aims to identify immune-related proteins expressed in beta-thal patients through autoantibody profiling using the Sengenics Immunome Array. This platform has been widely used to profile autoantibody responses in various types of diseases, including cancers [17,18], Parkinson’s disease [19] and infectious diseases [15,16]. The differential expression of immune-related proteins identified in beta-thal patients could provide insights into the potential pathways of disease progression, and hence, in future, better disease management can be undertaken through novel drug discovery. 

In this pilot study, we aimed to evaluate the immunome protein microarray, which utilizes the KREX technology to identify autoantibody signatures that could potentially be involved in the pathogenesis of beta-thal major.

## 2. Results

### 2.1. Patients

We collected serum from nine diagnosed cases of beta-thal major patients on regular blood transfusion. All patients had high bilirubin levels, and the majority had low hemoglobin levels. The most common mutation in these patients was β-codon 41/42 [-TTCT], and HCLS1 was identified in the majority of cases (Table 1).

### 2.2. Quality Control

All samples passed the quality control (QC); a variation of the signal the accepted threshold herein. The IgG controls coefficient of the variant (CV%) is 6.2 %, a signal of all proteins across samples and arrays has a CV% of 5.9 %. To assess the binding capacity of the fluorescent-conjugated secondary antibody, we used the serial dilution of IgG as a positive control. The benchmark for the confirmation of labelling efficiency and for spot detection was the accurate dilution series that passed quality control thresholds were used. The following Equation (1) represent the experimental ratios for the IgG dilution series.

(1) IgG1:IgG2:IgG3:IgG4:IgG5:IgG6x:0.5x:0.25x:0.125x:0.0625x:0.03125x

**Figure 1:**
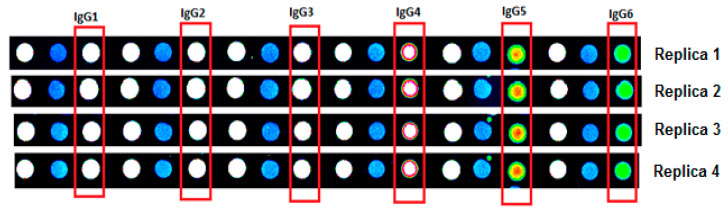
Sengenics-Immunome™ Protein Array quadruplicate images for IgG control spots showing serial dilution (Left to right).

**Figure 2:**
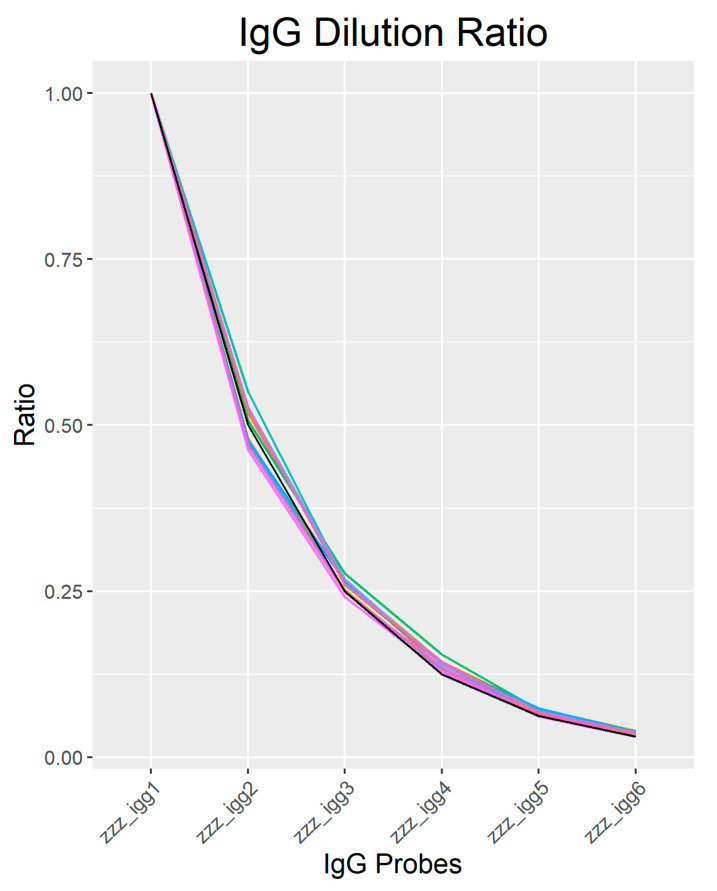
Plot of IgG 1–6 ratios for all samples (samples are represented in different colours) and the experimental dilution (Black line) as described in Equation 1. The mean coefficient of the variant (CV%) of each dilution to the experimental dilution across all samples was 6.207%.

The initial concentration of IgG spot is represented by x in the equation. The image of replica IgG spots in a serial dilution on the immunome slide are shown in Figure 1. Based on the signal intensity of IgG controls, secondary incubation was carried out with steps of serial dilution starting from IgG1 to IgG6. Variation studies of our IgG serial dilution to the experimental (ideal) IgG serial dilution can be seen in Figure 2. The CV% between each sample’s dilution to the experimental dilution was calculated, and the mean is 6.207%. 

**Figure 3:**
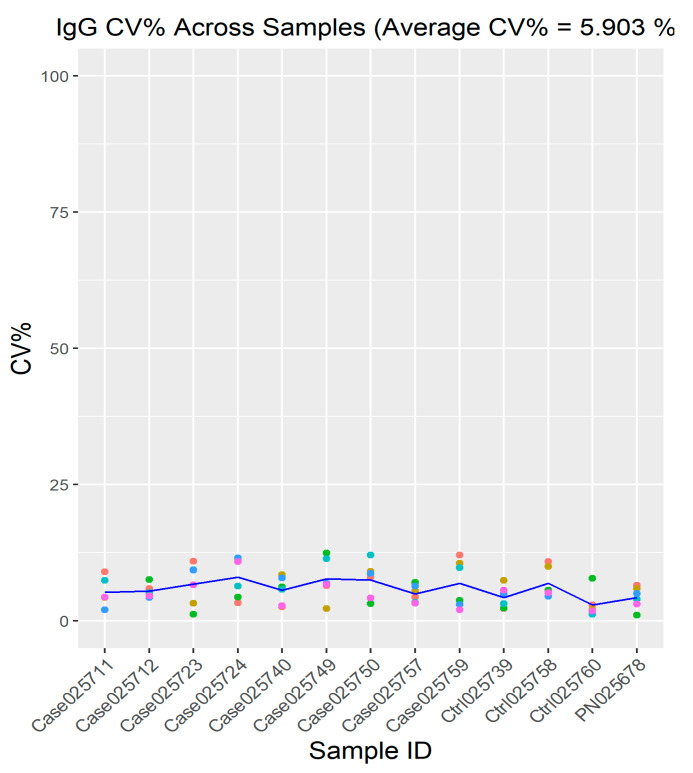
Intra-protein, intra-slide and inter-array CV% of six IgG replica spots across all samples. The mean CV% for all samples was found to be 5.903%. Blue line represents the mean CV% for each sample.

For the determination of the quality of the spot intensity, CV% between the replica arrays was used. It enabled the review of the IgG replica spot variability in each sample (Figure 3). For the present study, the mean of CV% for the IgG replica spots across all samples was 5.903%.

### 2.3. Cy3-BSA as Internal Control Showed Consistent Intensity across Samples

**Figure 4:**
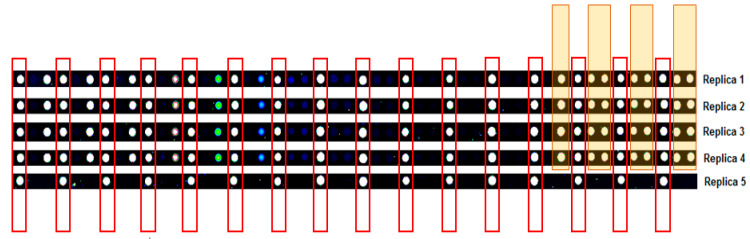
Sengenics-Immunome™ Protein Array replica images for all replicas of Cy3BSA control spots showing consistency in the intensities.

**Figure 5:**
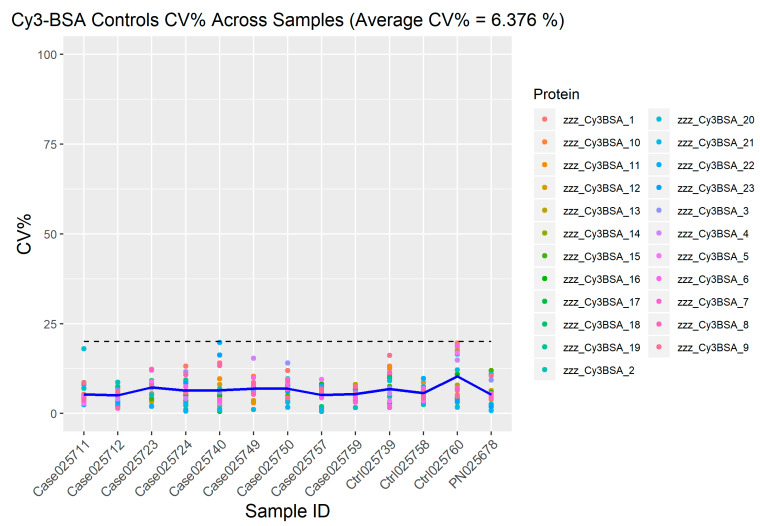
Intra-protein, intra-slide and inter-array CV% of six IgG replica spots across all samples. The relative fluorescence unit (RFU) mean CV% for all Cy3-BSA replicates across all samples was 6.376%; was below the cut-off points which was 15%. Blue line represents the mean CV% for each sample.

**Figure 6:**
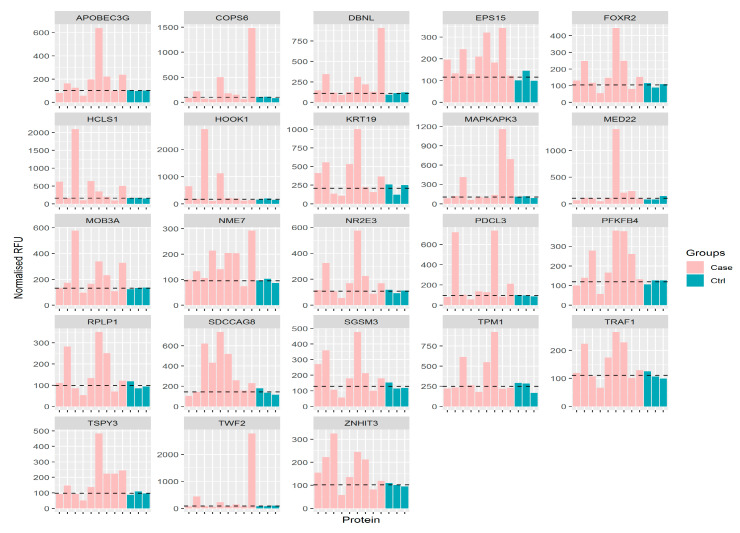
Profile of all 23 biomarkers in all beta-thal major (pink) in relative to healthy control (green). The discontinuous line represents the mean of control sample.

Cy3-BSA was included as a positive control in each array slide. Throughout the experiment, the concentration of Cy3-BSA replicas was kept constant as this served as a housekeeping probe for the normalization of signal intensities. Figure 4 shows an image of all replica Cy3BSA spots on the immunome slide. The raw mean and normalized mean relative fluorescence units (RFUs) of all Cy3-BSA controls across all samples in this study can be seen in Figure 5 and Figure 6, respectively. Figure 5 reflects the successful calibration of our RFU from this protein array experiment across all samples. Post-normalization, the Cy3-BSA controls show a common intensity value across all samples as depicted in Figure 4 and thus improving the quality of the data which allows a meaningful comparison to be made between different slides and/or experiments. The CV% between the mean RFU of all Cy3-BSA replicates across all samples was calculated, and the mean is 8.07%.

**Figure 7:**
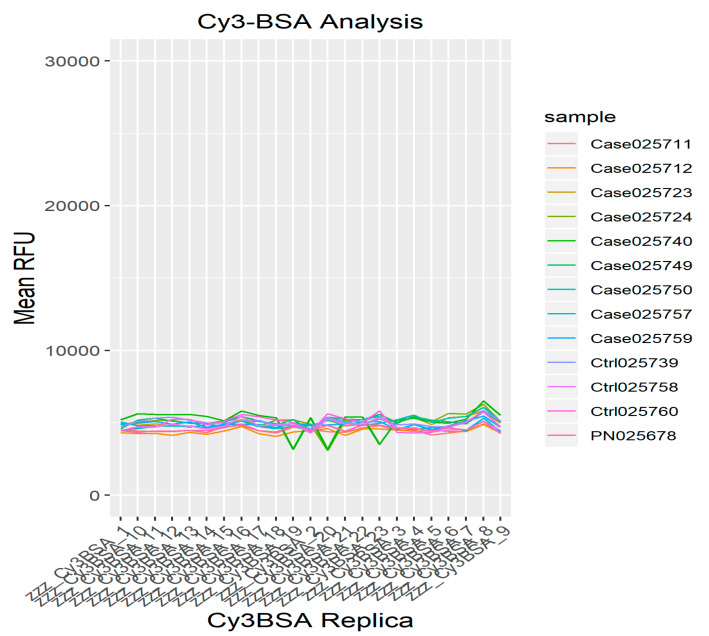
The RFU mean CV% for all Cy3-BSA replicates across all samples was 8.07%; was below the cut-off points which was 15%. Line plot of controls Cy3-BSA post-normalization clearly showed common intensities of Cy3BSA controls across all samples (samples are represented in different colors).

For the determination of the quality of the spot intensity, CV% between the replica arrays were used. It enabled review of variability in Cy3-BSA replica spots in each sample (Figure 7). In this study, the mean of CV% for the Cy3-BSA replica spots across all samples was 6.376%. 

### 2.4. Putative Biomarkers

A total of 23 putative biomarkers were identified in all beta-thal major patients (Table 2). A putative biomarker was determined based on penetrance fold change ≥ twofold and penetrance frequency >10% of the cohort. Figure 6 showed that a clear elevation of all 23 biomarkers in beta-thal major patients. Amongst the 23 putative biomarkers, TWF2 showed the highest penetrance fold-change (13.38-fold), while the lowest is TRAF1 (2.16-fold). This data represents a novel set of biomarkers that are based on autoantibody response screened in beta-thal patients. 

### 2.5. Heatmap

**Figure 8:**
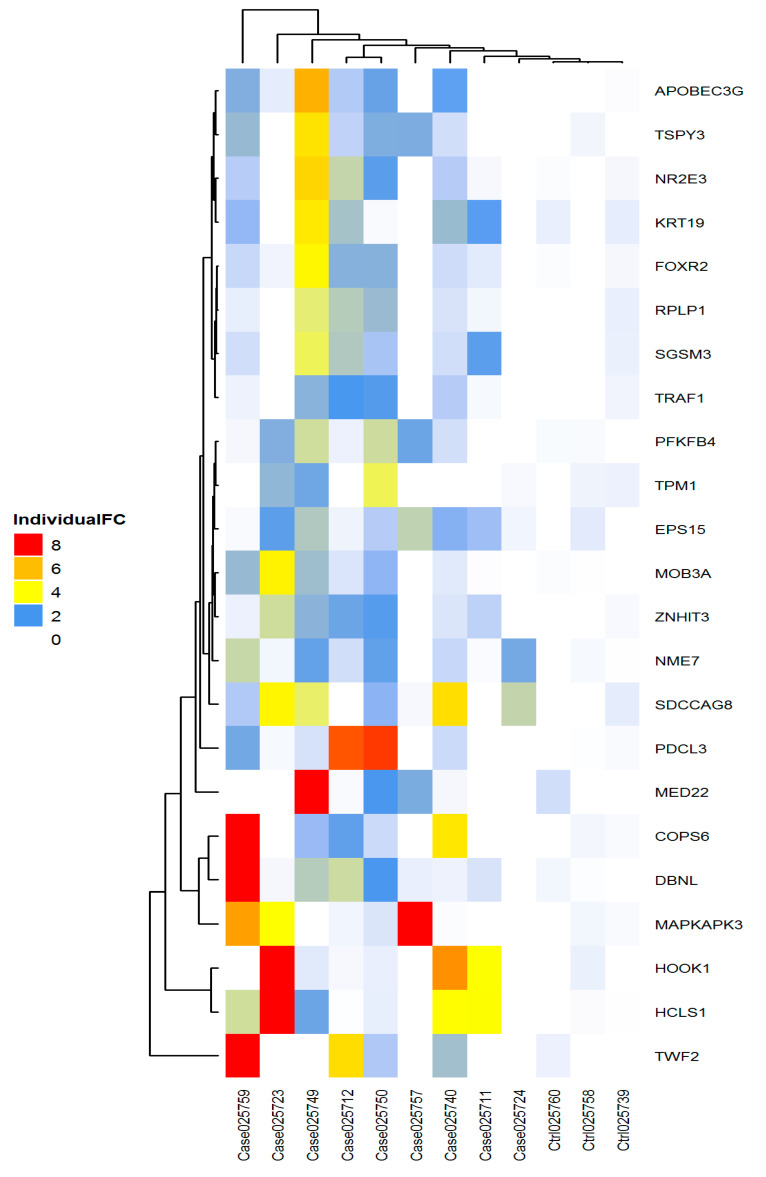
Stratification between beta-thal major (*n* = 9) and controls (*n* = 3). Unsupervised clustering was generated based on individual fold change value.

A heatmap analysis showed there was a clear stratification between beta-thal patients and healthy controls. Unsupervised clustering was generated based on individual fold change value of each biomarker in each patient, relative to the healthy control, as shown in Figure 8. However, there was no clear stratification between the patients themselves, which might be due to the small sample size. Nonetheless, autoantibody biomarkers were able to produce signature between control and patients.

### 2.6. Autoantibody Signature Based on Malay Ethnicity Cases

**Figure 9:**
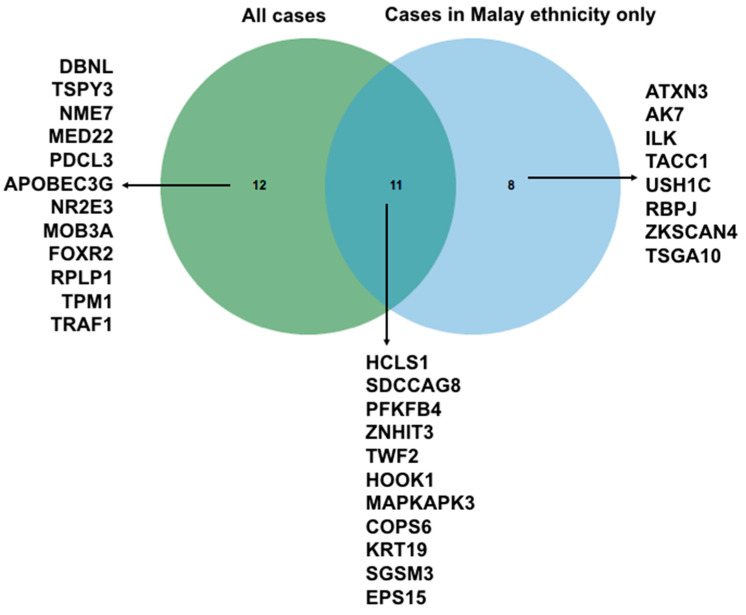
Venn diagram showed that there is a unique cluster of biomarkers of cases across two ethnicities of tested sample.

To determine whether ethnicity gives a different autoantibody signature, we removed Chinese cases (*n* = 3) and re-ran the penetrance fold change analysis. Here, we detect 19 autoantibodies specific to this sub-cohort (Figure 9), 11 of which overlap with the signature of combined all case samples (*n* = 9). A signature of 9 autoantibodies is shown unique to Malay ethnicities in this study (*n* = 6). To validate this signature, a larger cohort is needed. 

### 2.7. Autoantibody Biomarkers Showed Relevance to Thalassaemia Major Diseases

To identify the biological relevance of the shortlisted biomarkers, we subjected all 23 biomarkers to Open Target Platform (https://www.targetvalidation.org/), and the output is presented in Table 3. As shown (Table 3), 4 out of the 23 biomarkers are related to thalassemia subtypes, suggesting that there is a potential association between autoantibody with disease stratification, or perhaps if tested in a larger cohort, to disease severity. Although the limitation in this study is the sample size, this preliminary analysis data showed a potential use of autoantibody as a prognosis marker in beta-thal major.

### 2.8. Clustering of Biomarkers

**Figure 10:**
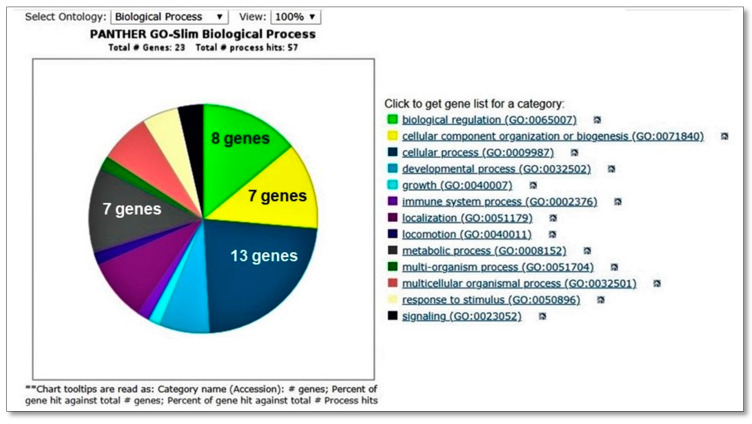
Pie-chart showing major biological processes that the differentially expressed proteins can be mapped to the analysis was performed using the Panther Classification system (http://www.pantherdb.org/).

When the biomarkers listed in Table 2 were analyzed using an online bioinformatics software, a Panther Classification system (http://www.pantherdb.org/), the analysis showed that most of the biomarkers could be clustered within four biological processes (Figure 10). These were (i) Biological regulation (HCLS1, TWF2, NR2E3, APOBEC3G, DBNL, SGSM3, MAPKAPK3, TRAF1); (ii) Cellular component organization or biogenesis (ZNHIT3, HCLS1, TWF2, DBNL, SDCCAG8, TPM1, HOOK1); (iii) Cellular process (ZNHIT3, HCLS1, TWF2, RPLP1, PFKFB4, NR2E3, APOBEC3G, DBNL, SDCCAG8, MAPKAPK3, TPM1, HOOK1, TRAF1) and (iv) Metabolic process (ZNHIT3, RPLP1, PFKFB4, APOBEC3G, NR2E3, MAPKAPK3, TRAF1).

## 3. Discussion

The primary causes of complications and mortality in beta-thal major patients are infections and immune abnormalities. Repeated blood transfusions result in immune disorders, which can be quantitative or qualitative [21]. This could be explained by the fact that immune regulating genes reside close to the hemoglobin β-chain locus [11]. Association of thalassemia and autoimmune disease also shows the reduced expression of hemorphins, which are endogenous atypical opioid peptides, generated by enzymatic hydrolysis of hemoglobin [22]. Reduced levels of hemorphins can cause vulnerability to autoimmunity [11]. 

The abnormalities of the immune system may be involved in organ dysfunction and have a possible role in the clinical course of beta-thal major. The elucidation of this association could help to explore new treatment modalities and improve the management of beta-thal major patients [23]. Altered lymphocyte subpopulations in beta-thal major may result in the abnormal concentration of regulatory T-cells [24,25], hence the negative response against foreign and self-antigens, [26] which warrants further investigations.

Chronic blood transfusions are associated with the risk of allo-sensitization and repeated infections. Studies suggest that chronic activation of the immune system is associated with iron overload and chelation therapy [27]. However, the risk of allo-sensitization can be reduced if the blood transfusion was started early in patients with hemoglobinopathies. The reason for this could be immune tolerance to allogenic RBC antigens when it was compared with patients who had transfusion later in life [28]. In the present study, all patients included had their first blood transfusion at less than one year of age. Furthermore, our patients were transfused regularly twice in a month. However, the relationship between the number of units transfused and allo-sensitization is still unknown in thalassemia [29].

There are still no standardized guidelines for the analysis of autoantibodies in thalassemia. Using the immunome protein microarray approach, we successfully identified 23 autoantibodies as potential biomarkers with individual sensitivity and specificity. Four biomarkers are known to be associated with thalassemia as shown in Table 3.

This study profiled the autoantibody response in beta-thal patients’ sera on an immunome array containing 1600+ full-length correctly folded, immune-related human antigens. Since >90% epitopes antibody epitopes are thought to be discontinuous and presented on the solvent-exposed surface of the target antigen [30], the interaction between plasma autoantibodies with correctly folded antigens on our array platform will provide increased specificity and sensitivity in autoantibody biomarker detection, by identifying true positives, whilst minimizing false positives and false negatives. We hypothesize that machine learning approaches will enable us to combine the resultant autoantibody signature with detailed clinical annotation provided in the tested samples, that could correlate with the severity of beta-thal. The KREX array-based serological assay is quantitative across five orders of magnitude, with detection limits in the pg/ml range. The autoantibody profiling was conducted using an autoantibody assay that was previously utilized with high reproducibility and high repeatability in various autoimmune, cancer and neurological diseases such as lupus [31], rheumatoid arthritis [32], Graves’ disease [33] Sjögren syndrome [34] and autoimmune hepatitis [35]. Autoantibodies, in principle, are detectable many years before clinical manifestation or symptoms. For instance, autoantibody biomarkers in a highly heterogeneous systemic sclerosis have been detected in patients’ sera as early as 27 years before clinical diagnosis [36]. Due to the sensitivity of detection at an asymptomatic stage, numerous studies on various types of cancers, including non-small cell lung cancer [37], breast cancer [38,39], kidney cancer [40] and melanoma [17] have employed autoantibodies screening for early detection purposes. Autoantibody specific profiles have also been identified in highly heterogeneous diseases such as autism [41] and arthritis [32,42], thus suggesting novel autoantibodies as plausible biomarkers for early diagnosis of a broader spectrum of diseases. Furthermore, due to the high sensitivity and half-life stability, autoantibody profiling can be used to stratify patients based on disease trajectory, such as the Sengenics Immunome protein array, to carry out serological studies in various cancers and autoimmune diseases. For example, in a study on a cohort of systemic lupus erythematous (SLE) patients (*n* = 277) and age-matched controls (*n* = 280), we identified 68 novel autoantigens, in addition to 11 known autoantigens, including the well-known SLE autoantigens TROVE2 and SSB [43]. Unsupervised cluster analysis revealed four distinct SLE clusters, which correlated, in part, with anti-nuclear antibody and anti-dsDNA autoantibody titers and which plausibly represent distinct molecular sub-types of SLE, with different disease trajectories and different responses to treatment.

Interestingly, 19 of the 23 identified markers are novel and not known previously for their association with thalassemia. Autoantibodies to these 23 proteins were identified to be present in the serum of beta-thalassemia major patients when compared to non-thalassemia controls. The evaluation of autoantibody signatures from differentially-reactive antigens associated with beta-thal major could help us to understand the disease pathophysiology. The majority of the identified biomarkers such as hematopoietic lineage cell-specific protein (HCLS1), Epidermal growth factor receptor substrate 15 (EPS15), drebrin like (DBNL), zinc finger HIT-type containing 3 (ZNHIT3), twinfilin actin-binding protein 2 (TWF2), Tropomyosin 1 (TPM1) [44] are associated with cell growth regulation and apoptosis [45]. Up-regulated autoantibodies may cause red blood cells to be susceptible to apoptosis with these proteins, which may increase disease severity and the need for a transfusion.

Comparing the available patient data with the biomarkers identified in this study, we found that β codon 41/42 [-TTCT] was the most common mutation, which is a frequent mutation type in Chinese β-thal carriers [46]. The penetrance frequency and fold changes of HCLS1 were high in these cases. Previously, it has been observed to be upregulated in several cancers, and its expression level is associated with increased cell migration, metastasis, and poor prognosis [47]. However, we cannot find its association with beta-thal major patients in literature; in the future, this association needs to be explored with a large dataset.

Another important group of proteins identified can be grouped under the actin-binding protein (ABP) group. The cytoskeleton of eukaryotic cells comprises actin, which plays an essential role in the maintenance of intracellular transport, morphology of cells, cellular movement, cell division and endocytosis. ABPs also regulate the organization of actin filaments in cells by higher-order cellular networking [48,49]. In this regard, autoantibodies directed against four proteins (HCLS1, DBNL, TWF2 and TPM1) were found to be elevated in beta-thal patients compared to controls. 

Disease-specific immunoreactivity or antibody profiles may provide insights into autoantibody signatures for the prognosis of beta-thal major. We found that four of the biomarkers associated with the thalassemia disease subtype can be further explored for disease stratification based on severity.

## 4. Materials and Methods

This study is approved by Medical Research and Ethics Committee (MREC) of the Malaysian Ministry of Health [KKM/NIHSEC/P19-618(11)] (9 July 2019) and the Research and Ethics Committee of the International Medical University, Malaysia (4.3/JCM-174/2019) (23 January 2019). This study is also registered with the National Medical Research Register (NMRR) of Malaysia (Research ID: 46698) (9 July 2019).

### 4.1. Study Design

The Sengenics Immunome Protein Array platform was used for high-throughput quantification of autoantibodies in 12 serum samples collected from nine (9) beta-thal Major patients and three (3) non-thalassemia controls, which were run together with two pooled normal sera (Sengenics Internal QC samples). The serum from the nine (9) beta-thal major was from six (6) male (4 Malays, 2 Chinese) and three (3) female (2 Malays and 1 Chinese) patients aged between 10 to 17 years old. The three non-thalassemia controls are males aged between 8 to 14 years old (2 Malay and 1 others). The serum samples were stored at −80 °C before analysis.

### 4.2. Serum/Plasma Dilution

The serum samples were placed in a shaking incubator set at 20 °C to allow thawing for 30 min. When completely thawed, each sample was vortexed vigorously three times at full speed and spun down for 3 min at 13,000 rpm using a microcentrifuge. Then, 22.5 µL of the sample was pipetted into 4.5 mL of Serum Assay Buffer (SAB) containing 0.1 *v*/*v* Triton, 0.1% *w*/*v* BSA in PBS (20 °C) and vortexed to mix three times. The tube was tilted during aspiration to ensure that the sera were sampled from below the lipid layer at the top. This serum/plasma dilution process was carried out in a class II biological safety cabinet. 

### 4.3. Biomarker Assay

The KREX array-based serological assays are quantitative across 5 orders of magnitude, with detection limits in the pg/mL range. KREX is known as world’s only technology that produces correct folding of functional full-length proteins. Using KREX, more than 98% of soluble proteins achieve correct folding, whereas the success rate of conventional approaches is only 48%. This assay entails serial dilution studies to be performed on patients’ serum prior to autoantibody assay on immunome array to ensure both assay and samples meet the accepted threshold, as outlined in quality control. 

The protein array slide was washed following the standard KREX protocol. All slides were scanned using the barcode scanner into the relevant batch record and incubated on a horizontal shaker at 50 rpm for 2 h at 20 °C.

### 4.4. Array Washing after Serum Binding

The slide was rinsed with SAB buffer, followed by shaking of slide box containing SAB buffer at 50 rpm at room temperature for 20 min. All slides were transferred sequentially and in the same orientation. 

### 4.5. Incubation with Cy3-anti IgG

After incubation, SAB buffer was used for dipping of slide at room temperature followed by removal of excess buffer with pure water for a few minutes. The slide was dried and then stored at room temperature until scanning. Agilent scanner (microarray laser scanner) at 10µm resolution was used for measurement of hybridization signals and fluorescence intensities were quantified as per manufacturer’s protocol. The Agilent Feature Extraction software was used for plotting of each spot. 

### 4.6. Bioinformatics Analysis

#### Image Analysis: Raw Data Extraction

The aim of the image analysis is to evaluate the amount of autoantibody present in the serum sample by measuring the median intensities of all the pixels within each probed spot. A raw .tiff format image file is generated for each slide. Automatic extraction and quantification of each spot on the array were performed using the GenePix Pro 7 software (Molecular Devices, CA, USA) which outputs the statistics for each probed spot on the array. This includes the mean and median of the pixel intensities within a spot along with its local background. A GAL (GenePix Array List) file for the array is generated to aid with image analysis. This file contains the information of all probed spots and their positions on the array. Following data extraction, a GenePix Results (.GPR) file was generated for each slide which contains the information for each spot: Protein ID, protein name and foreground and background intensities. In the datasheet generated from the experiment, both foreground and background intensities of each spot are represented as relative fluorescence units (RFUs). 

### 4.7. Data Handling and Pre-Processing

For each slide, proteins and control probes are spotted in quadruplicate—4 arrays on each slide. The following steps were performed to verify the quality of the protein array data before proceeding with data analysis. First, the calculation of net intensities for each spot was determined by subtracting background signal intensities from the foreground signal intensities of each spot. For each spot, the background signal intensity was calculated using a circular region with three times the diameter of the spot, centered on the spot. Next, the calculation of the percentage of the coefficient of the variant (CV%) was performed to determine the variations between the replica spots on each slide, based on Equation (2) below:

(2) $$CV\;\%=\frac{S.D}{Mean}\times 100\%$$

After this, we normalized the intensity using composite normalization that is quantile-based and total intensity-based modules. Bolstad’s alogrithm was used for the quantile module [50]. This allows the Cy3BSA control probes to detect and handle the flagged spots. The scaling factor for each sample was then obtained by intensity-based module. 

Quantile-Based Normalization of all cy3BSA across all samples were calculated based on the following equation:

(*i* = spot number and *j* = sample number)
Load all Cy3-BSA across all samples, *j*, into an *i* X *j* matrix X.
Sort spot intensities in each column *j* of X to *get X_sort_*
Take the mean across each row *i* of *X_sort_* to get < *X_i_* >
The intensity-based normalization on each sample was calculated based on the Equation (3) below:
Calculate the sum of the mean across each row *i,* Σ < *Xi* >
For each sample, *k,* calculate the sum of all Cy3-BSA controls, Σ *Xk*
For each sample, *k*,

(3) $$Scaling\;factor\;\left(k\right)=\frac{\;\Sigma \;<Xi>}{\Sigma {}^{Xk}}$$

### 4.8. Data Analysis

In this analysis, individual fold changes for both case and control were calculated as indicated in Equation (4) below:

(4) $$Individual\;FC=\frac{H_{Case\;or\;Control}}{\;\mu \;(H_{Control.})}$$

Those proteins with an individual fold change of less than 2-fold above the background threshold, their signal intensities (RFU) were replaced with zeroes. Next, penetrance frequency (number of case and control samples with individual fold changes -≥ 2-fold) for both case (*Frequency_case_)* and control *(Frequency_control_)* were determined for each protein along with their difference, as indicated in Equations (5)–(7).

(5) $$Frequency_{Case\;=\;n\;\left(Individual\;FC\;\left(Case\right)\right)\geq 2)}$$

(6) $$Frequency_{Control\;=\;n\;\left(Individual\;FC\;\left(Control\right)\right)\geq 2)}$$

(7) $$Frequency_{diff=Frequency_{Case}-Frequency_{Control}}$$

Penetrance fold changes for both case and control groups are calculated for each protein in Equations (8) and (9):

(8) $$Penetrance\;fold\;change_{case}=\frac{\mu (H_{Case}\left[i\right])}{\mu \left(H_{Control}\right)}$$

(9) $$Penetrance\;fold\;change_{control}=\frac{\mu (H_{Control}\left[i\right])}{\mu \left(H_{Control}\right)}$$

where, $H_{Case}\left[i\right]=H_{Case}\;with\;FC\;Case\;\geq 2\;fold$ and $H_{Control}\left[i\right]=H_{Control}\;with\;FC\;Control\;\geq 2\;fold$.

Based on the penetrance fold change described above, putative biomarkers are identified and ranked according to the i- penetrance fold-change >2.0; ii-penetrance frequency_case_ ≥10% and penetrance frequency differential ≥ 10%.

### 4.9. Unsupervised Clustering on Normalised RFU for 23 Biomarkers across 12 Samples

The unsupervised clustering of top 19 biomarkers across all 12 samples was performed using a hierarchical clustering method, i.e., Ward’s method [51] and distance calculated based on Euclidean distance. The heatmap shown in Figure 8 was plotted using ComplexHeatmap v 1.20.0 package [52] from Bioconductor.

## 5. Conclusions

We reported that serum from beta thal major is associated with autoantibody response using the immunome protein microarray approach. Based on this pilot study, 19 out of 23 autoantigens identified are novel biomarkers and have never been associated with beta-thal major before. Hence, further validation on these biomarkers is required to attest their importance in the pathogenesis of beta-thal major. A study with a larger sample size is needed to validate the autoantibody signature identified in this study for disease prognosis and pathology. Furthermore, given that beta-thal major highly associated with beta-globin gene mutations, future studies should also take specific mutations into consideration.

## Figures and Tables

**Table 1:** Clinical data of beta-thalassemia major patients and summary of their autoantibody biomarkers profile.

Case ID	Hemoglobin	WBC Count	Total Bilirubin	Mutation Analysis	Biomarkers
	(g/dL)	(×10^9^ /L)	(mol/L)		
025757	9.1	6.1	41	β+ IVS 1-5 [G-C] β variant codon 26 [G-A]	MAPKAPK3, TSPY3, PFKFB4
025724	8.6	6.8	21	Not available	SDCCAG8, NME7
025712	12.4	8.9	15	Not available	DBNL, TWF2, PDCL3, NR2E3, ZNHIT3
025711	11.1	5.3	34	α SEA deletion α+ α-thal 3.7Kb deletion	HCLS1, HOOK1
025740	9.9	6.4	74	β+ IVS 1-5 [G>C] β variant Codon 26 (GAG>GTG)	HCLS1, SDCCAG8, HOOK1, COPS6
025723	11.7	7.8	44	β codon 41/42 [-TTCT] β variant codon 26 [G-A]	HCLS1. SDCCAG8, ZNHIT3, HOOK1, MAPKAPK3, MOB3A, PFKFB4, ZNHIT3
025749	8.1	5.4	99	β codon 41/42 [-TTCT]	SDCCAG8, TSPY3, PFKFB4, APOBEC3G, APOBEC3G NR2E3KRT19, FOXR2, RPLP1, SGSM3, HCLS1, DBNL, ZNHIT3, NME7
025759	9.6	15.4	81	β codon 41/42 [-TTCT] β+ -28 [A-G]	HCLS1, DBNL, NME7, TWF2, MAPKAPK3, COPS6, TSPY3
025750	9.0	10.2	44	β codon 41/42 [-TTCT] β codon 71/72 [+A]	PFKFB4, PDCL3, TPM1, DBNL, TSPY3, ZNHIT3, NME7

**Table 2:** A total of 23 biomarkers were identified in all cases (nine samples) in relative to healthy control (three samples). The biomarkers showed high autoantibody titers in all cases identified using the penetrance fold change-based method.

No.	Proteins	Penetrance Frequency (* Case)	Penetrance Fold Change (* Case)	Mean (Control)
1	HCLS1	5	5.33	157.83
2	DBNL	4	4.07	109.26
3	SDCCAG8	4	4.01	144.09
4	TSPY3	4	3.02	97.43
5	PFKFB4	4	2.7	119.35
6	ZNHIT3	4	2.46	102.29
7	NME7	4	2.39	95.36
8	TWF2	3	13.38	85.86
9	HOOK1	3	9.21	163.31
10	MAPKAPK3	3	7.21	104.63
11	COPS6	3	7.08	103.80
12	MED22	3	5.95	103.00
13	PDCL3	3	5.89	94.35
14	APOBEC3G	3	3.58	102.11
15	NR2E3	3	3.49	107.26
16	KRT19	3	3.32	209.80
17	MOB3A	3	3.17	130.69
18	FOXR2	3	3.00	104.71
19	RPLP1	3	2.97	99.18
20	SGSM3	3	2.85	129.13
21	TPM1	3	2.79	248.36
22	EPS15	3	2.61	115.74
23	TRAF1	3	2.16	110.63

* Case: beta-thal major patients.

**Table 3:** List of biomarkers that has an association with thalassemia diseases. A disease association of the 23 antigens showing elevated autoantibody responses was established based on a literature and data mining approach using the Open Targets Platform (https://www.targetvalidation.org/) [20].

Disease Full Name	No. of Associated Targets	All Targets
Thalassemia	4	EPS15 HCLS1 KRT19 TPM1
Beta-thal and related diseases	3	EPS15 HCLS1 TPM1
Beta-thal	2	HCLS1 TPM1
Beta-thal intermedia	1	HCLS1
Hereditary persistence of fetal Hb- beta-thal	1	EPS15
Delta-beta-thal	1	EPS15
Beta-thal associated with another Hb anomaly	1	EPS15
Beta-thal major	1	HCLS1
Alpha-thal	1	KRT19
Alpha-thal and related diseases	1	KRT19
